# Effects of Oral Exposure to Mn-Doped ZnS Quantum Dots on Intestinal Tract and Gut Microbiota in Mice

**DOI:** 10.3389/fphys.2021.657266

**Published:** 2021-07-06

**Authors:** Yanjie Yang, Ruixue Xia, Xiaomei Zhang, Xu Wang, Yuchen Zhou, Honggang Wang, Yu Feng, Shuangyu Lv, Shaoping Ji

**Affiliations:** Institute of Molecular Medicine, Henan Provincial Engineering Center for Tumor Molecular Medicine, School of Basic Medical Sciences, Henan University, Kaifeng, China

**Keywords:** Mn-doped ZnS QDs, oral administration, intestinal tract, gut microbiota, mice

## Abstract

Mn-doped ZnS quantum dots (QDs) with excellent optical properties have been explored in a wide range of fields. Their potential adverse effects on biological systems and human health should be evaluated before biological application. In the present study, we investigated the effect of Mn-doped ZnS QDs on the intestinal tract and gut microbiota structures at 2 h and 14 days (d) after 14 d repeated oral exposure in mice. Flame atomic absorption spectrophotometry (FAAS), histopathological examination, and transmission electron microscopy (TEM) were used to assess the absorption and toxicity of Mn-doped ZnS QDs on the intestinal tract. The 16S rRNA gene sequencing was used to evaluate the gut microbial communities. Mn-doped ZnS QDs did not accumulate in the duodenum, jejunum, ileum, or colon. The Zn content of feces was not significantly higher than in the control group. No major histological changes were found in these tissues. The intestinal microvilli remained regular, but swelling of mitochondria and endoplasmic reticulum was detected by TEM at 14 d after the last gavage. A total of 2,712 operational taxonomic units (OTUs) were generated. Mn-doped ZnS QDs treatment did not significantly change the α-diversity of Richness, Chao1, Shannon, and Simpson indexes. According to principal component analysis (PCA), Mn-doped ZnS QDs had no effect on the overall structure of the gut microbiota. No significant change occurred at the phylum level, while three genera were downregulated at 2 h and seven changed at 14 d after the last gavage. Our findings revealed that Mn-doped ZnS QDs had a little stimulation of the intestinal tract and gut microbiota, and oral administration may be a safe route for biological application (such as bioimaging and drug delivery).

## Introduction

Quantum dots (QDs) are semiconductor nanomaterials with unique optical properties, such as broad excitation and narrow emission spectra (Yaghini et al., 2018). The fluorescence self-quenching may limit QDs application, but doping could avoid this problem due to the substantial ensemble Stokes shift (Yang Y. et al., 2015). Mn-doped ZnS QDs with excellent optical properties have been explored in biosensing, detecting, imaging, and drug delivery (Bwatanglang et al., 2016; Yang et al., 2019). The widespread production and application inevitably cause the direct and indirect release of Mn-doped ZnS QDs and their by-products into the environment, which may lead to unpredictable health effects (Heim et al., 2015; Zhao et al., 2016). Therefore, it is necessary to evaluate the potential toxic effects of Mn-doped ZnS QDs to understand their potential adverse effects on biological systems and human health.

The preferred route for clinical compliance of nanoparticles is oral administration (Li et al., 2018a). Nanoparticles can be taken up by the intestinal tract, and their absorption increases with decreasing particle diameter (Loeschner et al., 2011). In addition to direct ingestion, a proportion of inhaled and intravenous injected particulate materials are removed via hepatic processing and biliary excretion, and those that enter the intestinal tract are excreted from the body as feces (Bergin and Witzmann, 2013; Yang et al., 2014). Thus, the characterization of the permeability and uptake of Mn-doped ZnS QDs in intestinal cells is indispensable before biological application.

The intestinal tract comprises structurally and functionally distinct regions, including digestive, absorptive, secretory, and protective functions (Bergin and Witzmann, 2013; Hassan et al., 2017). More than 100 trillion bacteria live in human digestive tracts and we collectively call them the gut microbiota (van den Brule et al., 2016; Li et al., 2018a). Microbial activity is essential for intestinal functions, and microbes can affect energy metabolism, nutritional digestion and absorption, vitamin synthesis, inflammatory reaction, and immune status (Bergin and Witzmann, 2013; van den Brule et al., 2016; Wilding et al., 2016; Chen et al., 2019). There is increasing recognition that the gut microbiota plays crucial roles in the maintenance of host health (Bergin and Witzmann, 2013; Li et al., 2018b; Chen et al., 2019). Structural changes of the gut microbiota are closely related to various diseases, such as metabolic disorders (obesity, insulin-resistant diabetes, and non-alcoholic fatty liver disease), inflammatory bowel disease (IBD), and even cancer (Bergin and Witzmann, 2013; van den Brule et al., 2016; Li et al., 2018a). The effects of nanoparticles on the gut microbiota have attracted attention recently (Li et al., 2018a). Therefore, what occurs when Mn-doped ZnS QDs meet gut microbiota is worthy of exploration.

The aim of the present study was to investigate the effect of Mn-doped ZnS QDs on the intestinal tract and gut microbiota structures after 14 days repeated oral exposure in mice. We observed the effects at 2 h and 14 d after the end of exposure. Flame atomic absorption spectrophotometry (FAAS) was used to assess intestinal absorption of Mn-doped ZnS QDs. Histopathological examination and transmission electron microscopy (TEM) were used to investigate the intestinal toxicity. Non-culture-based, next-generation sequencing techniques were used to further evaluate gut microbial communities.

## Materials and Methods

### Animals and Treatment

Male Kunming mice aged 6–8 weeks (23 ± 1 g) were provided by the Animal Center of Henan Province (Zhengzhou, China). The mice were housed (4 or 5/cage) in a controlled environment, 12 h light–dark cycle, 22 ± 1°C, 50–60% relative humidity, and food and water *ad libitum*. The study and protocol were approved by the Committee of Medical Ethics and Welfare for Experimental Animals of Henan University School of Medicine.

Mn-doped ZnS QDs were synthesized as described in our previous study (Yang Y. et al., 2015). The average diameter of Mn-doped ZnS QDs was 3.51 ± 0.15 nm according to TEM (JEM2100Plus, JEOL, Japan) (Supplementary Figure 1A). Fluorescence spectra were recorded on a Fluorolog-3 spectrofluorometer (HORIBA Scientific, Edison, NJ, United States) (Supplementary Figure 1B). The strong fluorescence centered at 592 nm and the excitation maximum centered at 338 nm. The Mn contents were found to be 0.56% and Mn contents 49.32% in Mn-doped ZnS QDs using ICP-MS (X SERIES2, Thermo Fisher Scientific, United States). Experimental animals were randomly divided into the control group (normal saline) and Mn-doped ZnS QD-treated group. The dose of 5 mg/kg Mn-doped ZnS QDs was used following our previous study (Yang Y. et al., 2015). Mice were gavaged consecutively for 14 d with a total volume of 100 μl. According to the literature (van den Brule et al., 2016; Li et al., 2018b), fresh feces were acquired from each animal at 2 h after the last gavage (10 animals per group; control, TC; Mn-doped ZnS QDs, TQD) for assessment of the effect of Mn-doped ZnS QDs on gut microbiota. For observation or recovery experiment, we collected feces at 14 d after exposure (9 animals per group; control, RC; Mn-doped ZnS QDs, RQD) after the last gavage. All fresh fecal samples were snap-frozen in liquid nitrogen and stored at −80°C for subsequently analysis. The mice were sacrificed, and we collected duodenum, jejunum, ileum, and colon samples. Specimens for histopathological examination were immediately fixed in 10% neutral buffered formalin, while specimens for TEM were immediately fixed in 3% glutaraldehyde, and specimens for Zn content analysis were stored at −20°C.

### Quantitative Analysis of Zn Content by FAAS

The collected duodenum, jejunum, ileum, colon, and fecal samples (∼50 mg) were digested in 1 ml of 70% nitric acid for 3 h at 90°C, 0.1 ml of perchloric acid was added, and samples were heated for a further 30 min. The digested solution was diluted to 5 ml with deionized water, and Zn content was detected using AA-6800 FAAS (Shimazu, Japan).

### Histopathological Examination

After 24 h fixation in 10% neutral buffered formalin, tissue pieces were dehydrated progressively in ethanol, cleaned in xylene, embedded in paraffin blocks, sliced into 5-μm sections, and stained with hematoxylin and eosin (HE). The morphology of the tissue was observed using the NI-U optical microscope (Nikon, Japan).

### Transmission Electron Microscopy

After 48 h fixation in 3% glutaraldehyde at 4°C, tissues were transferred into 1% osmium tetroxide for 1 h post-fixation and then dehydrated in concentrated ethanol, embedded in Epon 812, cut into ultrathin sections, stained with uranyl acetate and lead citrate, and observed using a JEM-2000EX transmission electron microscope (JEOL, Japan) with Amt V601 CCD.

### DNA Extraction and 16S rRNA Gene Sequencing

Total genomic DNA was extracted using QIAamp 96 PowerFecal QIAcube HT kit (Hilden, Germany). Quality and quantity of the extracted DNA were verified by agarose gel electrophoresis and NanoDrop 2000 UV-Vis Spectrophotometer (Thermo Fisher Scientific, Wilmington, DE, United States). DNA samples were diluted with sterile water to 1 ng/μl. Universal primers 343F (5′-TACGGRAGGCAGCAG-3′) and 798R (5′-AGGGTATCTAATCCT-3′) were used to amplify the V3–V4 variable regions of 16S rRNA genes by polymerase chain reaction (PCR). Amplicon quality was detected by gel electrophoresis, purified with AMPure XP beads (Agencourt), and amplified for another round of PCR. After purification, the final amplicon was quantified using Qubit dsDNA assay kit (Life Technologies, Carlsbad, CA, United States). Equal amounts of purified amplicon were pooled, and sequencing on the Illumina MiSeq platform (San Diego, CA, United States).

### Sequence Processing and Analysis

Raw sequence data were processed using Trimmomatic software (Bolger et al., 2014) to detect and cut off ambiguous bases (N). After trimming of low-quality sequences, paired-end reads were assembled using FLASH software based on the parameters of 10 bp of minimal overlapping, 200 bp of maximum overlapping, and 20% of maximum mismatch rate (Reyon et al., 2012). Sequences with ambiguous, homologous sequences, below 200 bases or chimera were abandoned (Caporaso et al., 2010). The data of each sample were clustered to generate operational taxonomic units (OTUs) using Vsearch software (version 2.4.2) based on 97% similarity cutoff (Edgar et al., 2011). The representative read (maximum richness) of each OTU was selected using QIIME package. All representative reads were annotated and blasted against Silva database (version 123) using RDP classifier with confidence threshold of 70% to obtain taxonomic information (Wang et al., 2007). The community compositions of each sample were counted at the level of phylum, class, order, family, and genus. The Richness, Chao1, Simpson, and Shannon indexes of α-diversity were calculated for each sample. Principal component analysis (PCA) was used to reveal the β-diversity.

### Statistical Analysis

The data of Zn content of the intestinal tract and feces are presented as the mean ± SEM. The unpaired *t-*test was used to test the difference between the two groups. The differences in bacteria between the groups were assessed by Kruskal–Wallis test. A value of *p* < 0.05 was considered statistically significant.

## Results

### Quantitative Analysis of Zn Content

**FIGURE 1:**
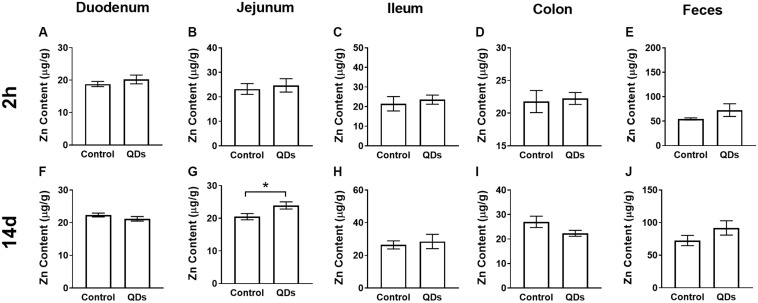
Quantitative analysis of Zn content at 2 h **(A–E)** and 14 d **(F–J)** after repeated oral administration of Mn-doped ZnS QDs for 14 days. **(A,F)** Duodenum, **(B,G)** Jejunum, **(C,H)** Ileum, **(D,I)** Colon, and **(E,J)** Feces. All data are presented as the mean ± SEM (*n* = 5–10). *p < 0.05 according to the unpaired *t-*test between the Mn-doped ZnS QD-treated and control groups.

The Zn content of the intestinal tract and fecal samples from the control group and mice gavaged for 14 d with Mn-doped ZnS QDs gavaged for 14 d mice were measured using FAAS (Figure 1). The Zn levels of duodenum, jejunum, ileum, and colon in mice treated with Mn-doped ZnS QDs were higher than that in the control group at 2 h after the last gavage, but not significantly (Figures 1A–D). At 14 d, the Zn content of jejunum samples was significantly increased compared with that in the control group (RC: 20.50 ± 0.96 μg/g; RQD: 23.93 ± 1.10 μg/g, *p* < 0.05, Figure 1G). The Zn contents of fecal samples collected at 2 h and 14 d were not significantly higher than those in the control group (Figures 1E,J).

### Histopathological Analysis

**FIGURE 2:**
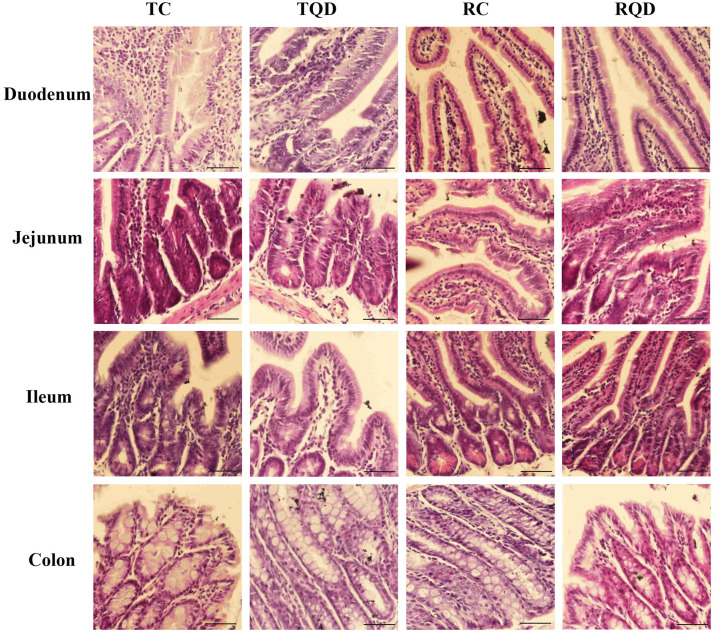
Representative hematoxylin and eosin staining of intestinal tract after repeated oral administration of Mn-doped ZnS QDs for 14 days. Scale bar: 50 μm.

The histopathology of intestinal tract tissues at 2 h and 14 d after Mn-doped ZnS QDs treatment are shown in Figure 2. Villi were well-conserved, and no obvious intestinal damage and structural alteration were observed in all samples of duodenum, jejunum, ileum, and colon.

### Ultrastructural Observation

**FIGURE 3:**
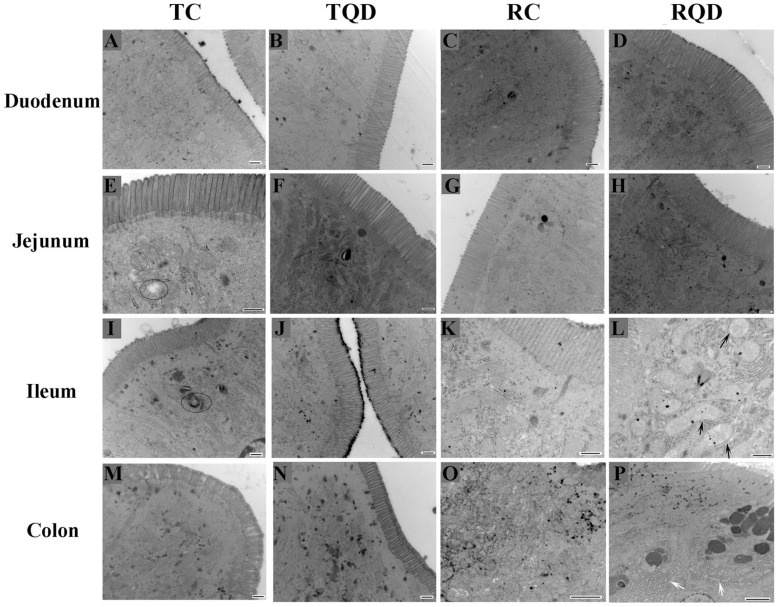
Ultrastructure of the intestinal microvilli of mice after repeated oral administration of Mn-doped ZnS QDs for 14 days. Black arrows: swelling of mitochondria; white arrows: swelling of endoplasmic reticulum; circles: autophagy. Scale bar: 500 nm for panels **(A–N)** and 2 μm for panels **(O–P)**.

To further determine the toxicity of Mn-doped ZnS QDs on the intestinal tract, the ultrastructure of the duodenum, jejunum, ileum, and colon was visualized by TEM. The microvilli of tissues remained regular in height, diameter, and spacing after oral administration of Mn-doped ZnS QDs (Figure 3). At 14 d after the last gavage, mitochondrial swelling was observed in the ileum (Figure 3L), while endoplasmic reticulum swelling occurred in the colon (Figure 3P).

### Effects of Mn-Doped ZnS QDs on Gut Microbiota

**FIGURE 4:**
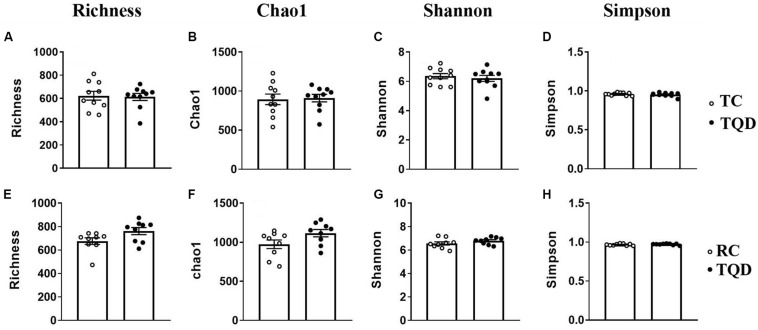
The α-diversity of the gut microbiota in mice at 2 h **(A–D)** and 14 d **(E–H)** after repeated oral exposure to Mn-doped ZnS QDs for 14 days. **(A,E)** Richness, **(B,F)** Chao1, **(C,G)** Shannon, and **(D,H)** Simpson. No significant difference was observed according to Kruskal–Wallis test (*n* = 9 or 10).

**FIGURE 5:**
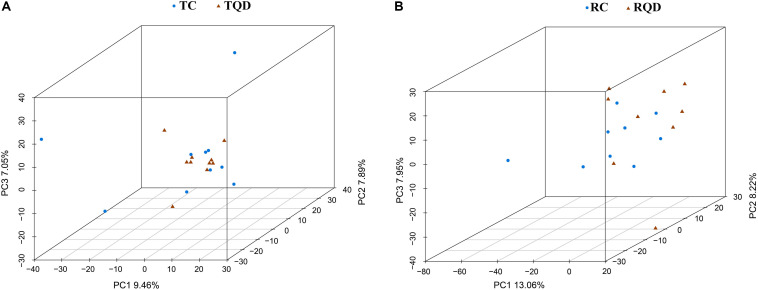
Principal component analysis to show the overall structures of the gut microbiota in mice after repeated oral exposure to Mn-doped ZnS QDs for 14 days. **(A)** 2 h and **(B)** 14 days.

After oral administration of 5 mg/kg Mn-doped ZnS QDs for 14 d, fresh feces were collected from each mouse at 2 h and 14 d after the last gavage, and microbial community profiles were constructed by 16S rRNA gene sequencing. We obtained 17,847–45,226 valid tags per sample, and sequences were subsampled to 14,277 for analysis. On the basis of 97% similarity, a total of 2,712 OTUs were generated, including 384–889 OTUs per sample. A summary of the sample tags and OTUs is shown in Supplementary Table 1. We calculated the α-diversity indexes of Richness, Chao1, Shannon, and Simpson indexes (Figure 4). Repeated oral dosing of Mn-doped ZnS QDs had no significant impact on all the above indexes at 2 h (Figures 4A–D) and 14 d (Figures 4E–H) after the last gavage. PCA was used to assess the effect of Mn-doped ZnS QDs on the overall structure of the gut microbiota (Figure 5). The results showed that Mn-doped ZnS QDs did not shift the microbial community structures at 2 h (Figure 5A). However, separation tendency between the Mn-doped ZnS QDs and control group was observed at 14 d (Figure 5B).

**FIGURE 6:**
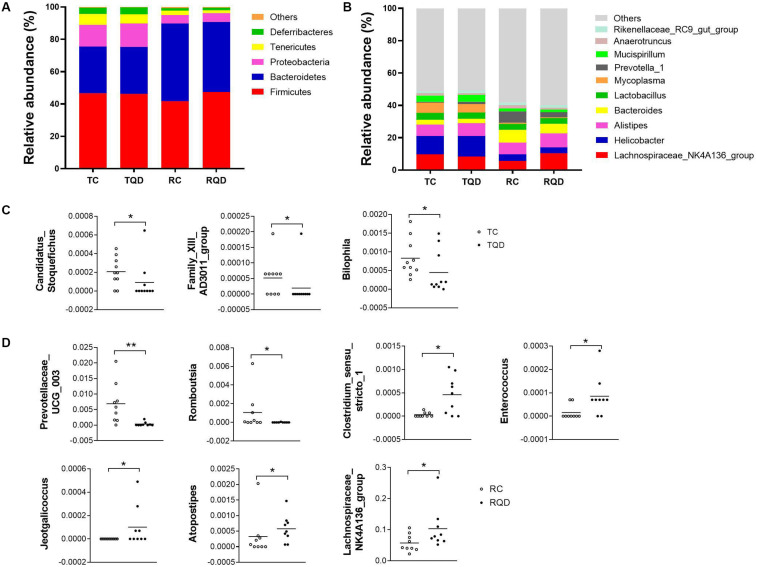
Abundance of **(A)** phyla and **(B)** genera in the gut microbiota after repeated oral exposure to Mn-doped ZnS QDs for 14 days. Scatter plots of significantly changed genera induced by Mn-doped ZnS QDs at 2 h **(C)** and 14 days **(D)** after the last gavage. *p < 0.05 and **p < 0.01 according to Kruskal–Wallis test (*n* = 9 or 10).

According to taxonomic assignment, the structure and composition of the gut microbiota communities at phylum and genus levels are shown in Figure 6. The top five phyla in the gut microbial communities were *Firmicutes*, *Bacteroidetes*, *Proteobacteria*, *Tenericutes*, and *Deferribacteres*, and >99% of the sequences were within the top three phyla (Figure 6A). There was no significant change in the above phyla at 2 h and 14 d after repeated oral exposure to Mn-doped ZnS QDs. At the genus level, the classification of bacteria tended to be dispersed. The 10 most abundant genera were *Lachnospiraceae NK4A136 group*, *Helicobacter*, *Alistipes*, *Bacteroides*, *Lactobacillus*, *Mycoplasma*, *Prevotella 1*, *Mucispirillum*, *Anaerotruncus*, and *Rikenellaceae RC9 gut group*, and we mapped 40–49% of the sequences (Figure 6B). Mn-doped ZnS QDs treatment significantly decreased the abundance of *Candidatus Stoquefichus* (*p* < 0.05), *Family XIII AD3011 group* (*p* < 0.05), and *Bilophila* (*p* < 0.05) at 2 h after the last gavage (Figure 6C). The changed genera increased to seven at 14 d after repeated oral exposure to Mn-doped ZnS QDs (Figure 6D): *Prevotellaceae UCG 003* (*p* < 0.01) and *Romboutsia* (*p* < 0.05) were decreased, while *Clostridium sensu stricto 1* (*p* < 0.05), *Enterococcus* (*p* < 0.05), *Jeotgalicoccus* (*p* < 0.05), *Atopostipes* (*p* < 0.05), and *Lachnospiraceae NK4A136* group (*p* < 0.05) were increased compared with the control group. It should be pointed out that the *Lachnospiraceae NK4A136 group* was the most abundant genus.

The community composition of gut microbiota at the level of class, order, and family is shown in Supplementary Figure 2. Mn-doped ZnS QDs treatment led to significant changes at the family level at 14 d after repeated oral administration (Supplementary Figure 3).

## Discussion

In this study, mice were subjected to repeated oral exposure to Mn-doped ZnS QDs for 14 d, and the toxicity in the intestinal tract and gut microbiota was assessed at 2 h and 14 d after the last gavage. The Zn contents of the duodenum, jejunum, ileum, and colon did not significantly increase at 2 h, indicating that Mn-doped ZnS QDs did not accumulate in these tissues. Li et al. reported that short-rod MSNs (mesoporous silica nanoparticles, AR = 1.75) and long-rod MSNs (AR = 5) did not induce obvious change of Si content in the intestine at 2 h after administration (Li et al., 2015). Loeschner et al. (2011) also reported that Ag nanoparticles were not located in the microvilli or within the enterocytes by TEM at 24 h after oral gavage twice a day for 28 days. Chronic oral exposure of γ-Fe_2_O_3_ nanoparticles did not accumulate in the duodenum, and the feces were the main excretion route (Chamorro et al., 2015). Our previous study also reported that QDs were primarily excreted in feces (Yang et al., 2014). However, in the present study, the Zn content in feces was higher than in the control group, although not significantly.

No major histological changes were found in the duodenum, jejunum, ileum, and colon. No abnormality of the microvilli was observed after Mn-doped ZnS QDs treatment, suggesting that Mn-doped ZnS QDs had a little stimulation on the intestinal tract. Our previous study showed that Mn-doped ZnS QDs did not cause obvious oxidative stress damage to the liver in mice (Yang Y. et al., 2015). However, swelling of mitochondria and endoplasmic reticulum was detected at 14 d after the last gavage. This result was in agreement with previous studies that CdTe QDs and QD705 induced mitochondrial swelling *in vitro* (Lin et al., 2012; Xiang et al., 2018). Nickel nanoparticles cause mitochondrial swelling and enlargement of the endoplasmic reticulum in ovarian tissues of female rats (Kong et al., 2016). Endoplasmic reticulum swelling was observed in the liver tissues and primary astrocytes after exposure to ZnO nanoparticles (Yang X. et al., 2015; Song et al., 2019).

Gene sequencing analysis of 16S rRNA was performed to disclose the variations of bacterial communities in feces. The α-diversity indexes of Richness (based on the number of OTUs) and Chao1 (based on the rare OTUs) are used to determine the richness in a community, while Shannon and Simpson indexes detect the richness and evenness of a community (van den Brule et al., 2016; Xia et al., 2017). Mn-doped ZnS QDs did not significantly change any of the above indexes, indicating that they had no effect on bacterial richness and diversity. According to the result of PCA, Mn-doped ZnS QDs could not markedly shift the overall structure of the gut microbiota at 2 h and 14 days. Taxon-based analysis also revealed a small change in the gut microbial composition by Mn-doped ZnS QDs treatment. We speculate that the small differences were due to the good biocompatibility of Mn-doped ZnS QDs.

Firmicutes and Bacteroidetes are the main phyla in mouse and human gut microbiota (Bergin and Witzmann, 2013; van den Brule et al., 2016). Mn-doped ZnS QDs treatment did not change their abundance and had no effect on other abundant phyla. Three genera were downregulated by Mn-doped ZnS QDs at 2 h, including *C. Stoquefichus*, *Family XIII AD3011 group*, and *Bilophila.* The *Family XIII AD3011 group* genus was detected in other studies, but is still poorly characterized (Guo et al., 2018; Yu et al., 2020). *Bilophila*, a sulfite-reducing bacterial genus, is associated with IBD (Haro et al., 2016; Santos-Marcos et al., 2018). At 14 d after the last gavage, the number of changed genera increased to seven (*Prevotellaceae UCG 003*, *Romboutsia*, *Clostridium sensu stricto 1*, *Enterococcus*, *Jeotgalicoccus*, *Atopostipes*, and *Lachnospiraceae NK4A136 group*). *Prevotellaceae UCG-003* belongs to a new bacterial family, and the increase in its abundance may exacerbate inflammation (Sun et al., 2019). *Prevotellaceae UCG-003* was significantly decreased after Mn-doped ZnS QDs treatment, which was similar to the IBD related to the genus *Bilophila*. Gerritsen et al. (2014) reported that the genus *Romboutsia* is characterized by the predominance of straight-chain saturated and unsaturated fatty acids (mainly C16 or C17), and absence of branched-chain fatty acids, dimethyl acetals, and aldehydes, but their function is scarce. The genus *Clostridium sensu stricto* is characterized by a wide biogeography and ability to inhabit anaerobic environments. Some *Clostridium sensu stricto* cause disease, while others produce added-value chemicals (Udaondo et al., 2017). The Gram-positive genus *Enterococcus* is the major causative agents of healthcare-associated infections (Garcia-Solache and Rice, 2019). The upregulation of *Clostridium sensu stricto* and *Enterococcus* by Mn-doped ZnS QDs increases the risk of disease. The genus *Jeotgalicoccus* is Gram-positive, non-motile, catalase- and oxidase-positive, and coccus-shaped (Martin et al., 2011). *Lachnospiraceae NK4A136 group*, the most abundant genus upregulated by Mn-doped ZnS QDs, is reported to be beneficial to intestinal function and decreased by oral administration of nickel (Zhou et al., 2019).

## Conclusion

In this study, the effect of Mn-doped ZnS QDs on the intestinal tract and gut microbiota was investigated in mice at 2 h and 14 d after repeated oral exposure. Mn-doped ZnS QDs did not accumulate in the duodenum, jejunum, ileum, and colon. There was no abnormity of villi and microvilli, but swelling of mitochondria and endoplasmic reticulum was detected in the ileum and colon at 14 days. According to gene sequencing analysis of 16S rRNA, Mn-doped ZnS QDs did not affect the richness, evenness, and structure of the microbial community. Mn-doped ZnS QDs induced small changes in the gut microbial composition according to taxon-based analysis. Our findings revealed that Mn-doped ZnS QDs had a small stimulus on the intestinal tract and gut microbiota.

## Data Availability Statement

The datasets presented in this study can be found in online repositories. The names of the repository/repositories and accession number(s) can be found below: BioProject (accession: PRJNA724902).

## Ethics Statement

The animal study was reviewed and approved by the Committee of Medical Ethics and Welfare for Experimental Animals of Henan University School of Medicine (Number: HUSOM2016-119).

## Author Contributions

YY, SL, and SJ designed the experiments and made data analysis. YY and RX performed the main experiments. XZ, XW, YZ, HW, and YF took part in part of the experiments. YY wrote the draft of the manuscript. SL and SJ contributed to revise the writing. All authors read and discussed part sections of the manuscript, and endorsed the final manuscript.

## Conflict of Interest

The authors declare that the research was conducted in the absence of any commercial or financial relationships that could be construed as a potential conflict of interest.

## Supplementary Material

Supplementary Figure 1 Characterization of Mn doped ZnS QDs.

Supplementary Figure 2 Abundance of class, order and family in the gut microbiota after repeated oral exposure to Mn doped ZnS QDs for 14 days.

Supplementary Figure 3 Scatter plots of significantly changed families induced by Mn-doped ZnS QDs at 14 d after the last gavage.

Supplementary Table 1 Summary of the sample tags and OTUs.

## Abbreviations

QDs: quantum dots
d: days
TEM: transmission electron microscopy
OTUs: operational taxonomic units
PCA: principal component analysis
FAAS: flame atomic absorption spectrophotometry
TC: control at 2 h
TQD: Mn-doped ZnS QDs at 2 h
RC: control at 14 d
RQD: Mn doped ZnS QDs at 14 d
HE: hematoxylin and eosin
MSNs: mesoporous silica nanoparticles.
