# Supplementary material for: Effects of Oral Exposure to Mn-Doped ZnS Quantum Dots on Intestinal Tract and Gut Microbiota in Mice
Source: Front Physiol. 2021 Jul 6;12:657266. doi: 10.3389/fphys.2021.657266 (PMC8290145; doi:10.3389/fphys.2021.657266)
Supplement: Supplementary Figure 1 — Characterization of Mn doped ZnS QDs. [file Data_Sheet_1.docx]

**Table S1** Summary of the sample tags and OTUs.

| **Sample** | **Clean Tags** | **Valid Tags** | **Valid Percent** | **OTU Counts** |
| --- | --- | --- | --- | --- |
| TC1 | 34618 | 30489 | 88.07% | 526 |
| TC2 | 37618 | 32497 | 86.39% | 489 |
| TC3 | 35867 | 31209 | 87.01% | 732 |
| TC4 | 33621 | 27250 | 81.05% | 559 |
| TC5 | 35199 | 32329 | 91.85% | 563 |
| TC6 | 41422 | 35881 | 86.62% | 638 |
| TC7 | 30383 | 26674 | 87.79% | 651 |
| TC8 | 41114 | 34625 | 84.22% | 802 |
| TC9 | 21356 | 19780 | 92.62% | 455 |
| TC10 | 23317 | 19709 | 84.53% | 716 |
| TQD1 | 25340 | 22500 | 88.79% | 384 |
| TQD2 | 25015 | 21669 | 86.62% | 631 |
| TQD3 | 27019 | 23501 | 86.98% | 625 |
| TQD4 | 27222 | 23193 | 85.20% | 645 |
| TQD5 | 22018 | 19308 | 87.69% | 612 |
| TQD6 | 24024 | 21007 | 87.44% | 696 |
| TQD7 | 50841 | 44247 | 87.03% | 642 |
| TQD8 | 43835 | 40585 | 92.59% | 535 |
| TQD9 | 46618 | 41222 | 88.43% | 615 |
| TQD10 | 41893 | 36592 | 87.35% | 677 |
| RC1 | 34510 | 32500 | 94.18% | 661 |
| RC2 | 51334 | 45226 | 88.10% | 682 |
| RC3 | 36320 | 32551 | 89.62% | 463 |
| RC4 | 50421 | 44276 | 87.81% | 707 |
| RC5 | 44039 | 41075 | 93.27% | 709 |
| RC6 | 50184 | 43822 | 87.32% | 722 |
| RC7 | 50179 | 44590 | 88.86% | 732 |
| RC8 | 46220 | 40494 | 87.61% | 668 |
| RC9 | 50616 | 43969 | 86.87% | 744 |
| RQD1 | 37702 | 32376 | 85.87% | 831 |
| RQD2 | 48145 | 42984 | 89.28% | 603 |
| RQD3 | 48070 | 41637 | 86.62% | 889 |
| RQD4 | 42767 | 37518 | 87.73% | 660 |
| RQD5 | 20940 | 17847 | 85.23% | 803 |
| RQD6 | 36611 | 32458 | 88.66% | 784 |
| RQD7 | 33337 | 29004 | 87.00% | 848 |
| RQD8 | 29104 | 25315 | 86.98% | 851 |
| RQD9 | 36156 | 31849 | 88.09% | 699 |





**Figure S1** Characterization of Mn doped ZnS QDs. (A) Transmission electron microscopy (TEM) and (B) photoluminescence (PL) spectra.

**
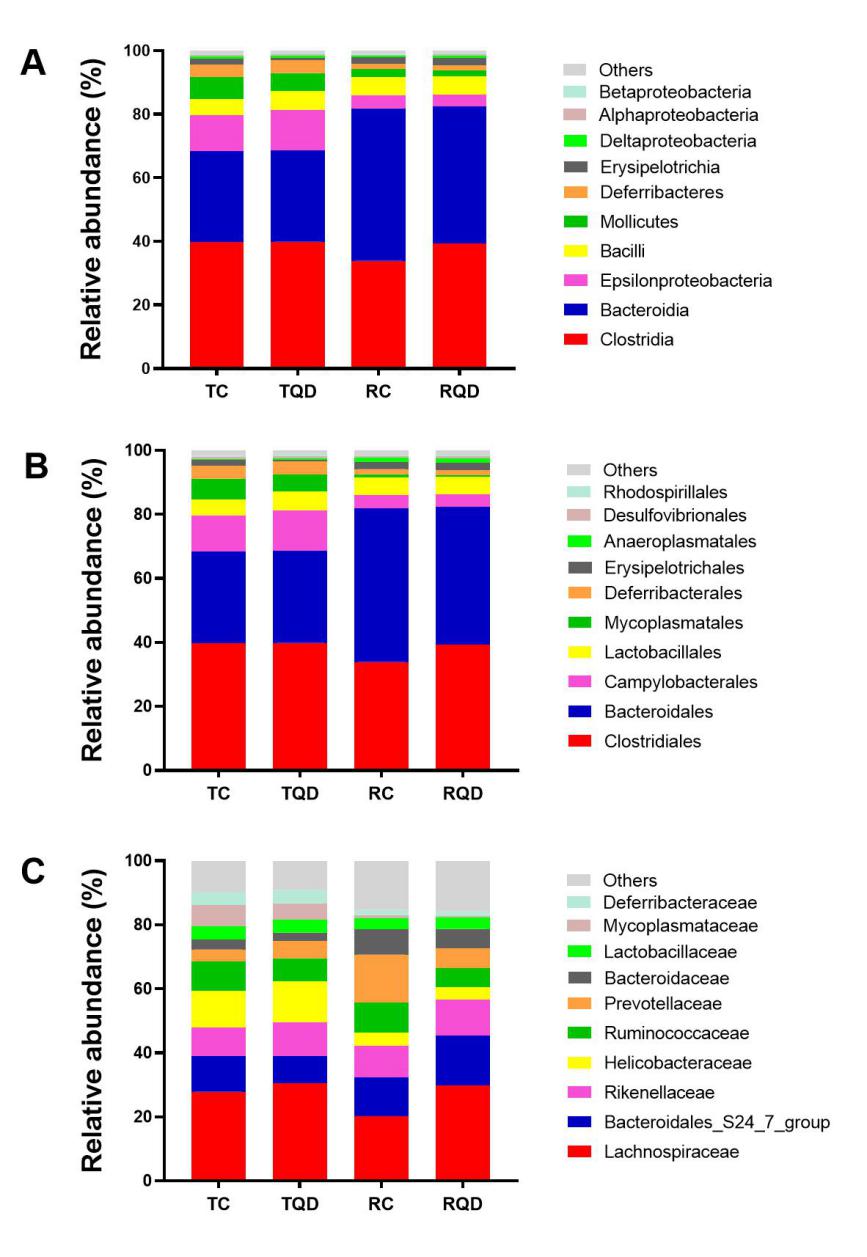
**

**Figure S2** Abundance of (A) class, (B) order and (C) family in the gut microbiota after repeated oral exposure to Mn doped ZnS QDs for 14 days.


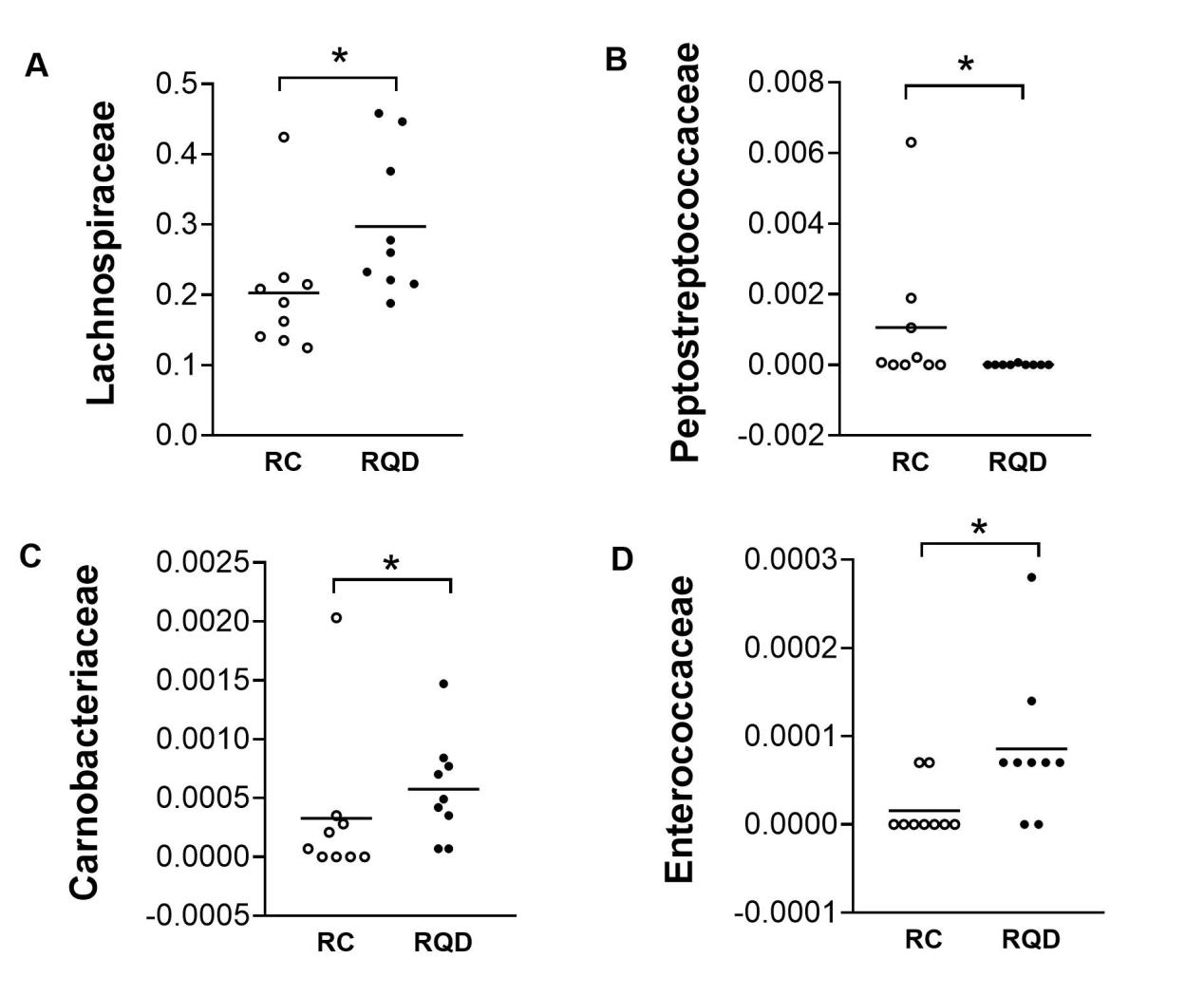


**Figure S3** Scatter plots of significantly changed families induced by Mn-doped ZnS QDs at 14 days after the last gavage. **p* < 0.05 according to Kruskal-wallis test (n = 9).
